# Impedance-Based Pre-Stress Monitoring of Rock Bolts Using a Piezoceramic-Based Smart Washer—A Feasibility Study

**DOI:** 10.3390/s17020250

**Published:** 2017-01-27

**Authors:** Bo Wang, Linsheng Huo, Dongdong Chen, Weijie Li, Gangbing Song

**Affiliations:** 1 Key Laboratory of Transportation Tunnel Engineering, Ministry of Education, Southwest Jiaotong University, Chengdu 610031, China; ahbowang@home.swjtu.edu.cn; 2 State Key Laboratory of Coastal and Offshore Engineering, Dalian University of Technology, Dalian 116024, China; lshuo@dlut.edu.cn (L.H.); chendongjt@163.com (D.C.); 3 Smart Material and Structure Laboratory, Department of Mechanical Engineering, University of Houston, Houston, TX 77204, USA; wli27@uh.edu

**Keywords:** rock bolt monitoring, pre-stress level monitoring, piezoceramic materials, smart washer, electro-mechanical impedance

## Abstract

Pre-stress degradation or looseness of rock bolts in mining or tunnel engineering threatens the stability and reliability of the structures. In this paper, an innovative piezoelectric device named a “smart washer” with the impedance method is proposed with the aim of developing a real-time device to monitor the pre-stress level of rock bolts. The proposed method was verified through tests on a rock bolt specimen. By applying high-frequency sweep excitations (typically >30 kHz) to the smart washer that was installed on the rock bolt specimen, we observed that the variation in impedance signatures indicated the rock bolt pre-stress status. With the degradation of rock bolt pre-stress, the frequency in the dominating peak of the real part of the electrical impedance signature increased. To quantify the effectiveness of the proposed technique, a normalized root mean square deviation (RMSD) index was developed to evaluate the degradation level of the rock bolt pre-stress. The experimental results demonstrated that the normalized RMSD-based looseness index, which was computed from the impedance value detected by the “smart washer”, increased with loss of the pre-stress of the rock bolt. Therefore, the proposed method can effectively detect the degradation of rock bolt pre-stress, as demonstrated by experiments.

## 1. Introduction

Thanks to their low cost, simplicity, and ease of installation, rock bolts, as a major measure to enhance the bearing capacity of surrounding rock and foundations, have been widely used in mining, tunnel, and geotechnical engineering [1]. Rock bolts are used as either temporary or permanent support systems to prevent the movement and expansion of rock strata. Rock bolts increase the stability of surrounding rock or soil by increase the cohesiveness and internal friction angle. However, for rock-bolted structures, in some cases, pre-tension degradation may lead to a compromised structure integrity or a reduction in load bearing capacity. Therefore, monitoring the pre-load status of rock bolts is essential in evaluating the health condition of a given rock bolt and its entire structure.

A considerable amount of research has been conducted to monitor rock bolt pre-tension status. All related research can be classified into two categories: the destructive testing method and the non-destructive testing method. A destructive testing method is a traditional way of diagnosing the working condition of a rock bolt and includes core drilling detection and the pull-out test method [2]. Core drilling detection is an outdated method that is rarely used now. The pull-out test is a method that can measure the bearing state of a specific rock bolt. The shortcoming of a destructive testing method is that the rock bolt will be destroyed. The guided wave propagation in a free bar was first studied numerically in the late nineteenth century by Pochhanmer [3] and Chree [4], which formed the foundation for guided wave-based damage detection methods. In recent years, due to its non-destructive nature, the ultrasonic guided wave method, as a non-destructive testing method, has gained popularity in rock bolt monitoring. A test method based on the frequency response of rock bolts was developed to determine encapsulation conditions [5]. The test determined the dominant frequency response when the rock bolt was struck with a hammer system attached to the bolt head. Beard et al. [6,7,8,9] proposed an ultrasonic pulse echo inspection technique, which was carried out from the free end of the rock bolt. The result showed that high frequency modes have low attenuation. A research group from Dalhousie University, Canada, consecutively investigated the influence of curing time [10], grouted length [11], grout strength [12], and missing grout [13] on the characteristics of ultrasonic guided waves on the grouted rock bolt. Another research group from the Republic of Korea also systematically conducted experiments and field tests on the rock bolt integrity monitoring using ultrasonic guided waves [14,15,16]. The wavelet transform was adopted to extract useful information on the grouting conditions of the rock bolt.

An electro-mechanical impedance (EMI)-based damage detection technique using lead–zirconate–titanate (PZT) patches is becoming a promising tool for detecting local damage in a wide variety of structures [17,18,19]. In this technique, a PZT patch is employed as both a sensor and an actuator. The impedance technique detects the variations of structural mechanical impedance caused by the occurrence of damage. According to the coupling theory of a PZT patch bonded on a host structure, the electrical impedance or admittance (inverse of impedance) of the PZT patches is directly related to the mechanical impedance of the host structure and will be affected by the presence of structural damage. Through monitoring the electrical impedance or admittance of the PZT patches bonded on the host structure and comparing it to a baseline measurement, the integrity of the host structure can be qualitatively determined. The small-sized PZT patches can be easily bonded on, or embedded into, a structure, even in inaccessible areas, to monitor the damage evolution of the host structure. This technique has the advantages of real-time and minimum requirements on transducers and data processing, which facilitates autonomous structural health monitoring. Research endeavors have been reported in applying the EMI technique for damage detection in a variety of structures, such as concrete strength monitoring [20,21,22], dental implant assessment [23,24], corrosion monitoring of reinforced concrete [25], lap-joint monitoring [26], and concrete-encased composite structures [27].

In this paper, we adopted and extended a newly developed technology, called a “smart washer”, which was fabricated by sandwiching a waterproof PZT patch with two pre-machined flat metal rings for bolted connection monitoring. Previous work has shown that it has great damage detection potential for bolted connections using the active sensing method. In this study, by integrating the piezoelectric impedance method with the smart washer, the pre-tension looseness of a rock bolt was monitored. To enable the experimental study, a special loading frame that can adjust the pre-tension of a rock bolt was designed and fabricated. The piezoceramic smart washer was installed between the nut and the anchor plate on the rock bolt. The smart washer functioned as an actuator and a sensor, so it generated stress waves that travel across the rock bolt and detect the stress waves that cross the specimen. The piezoelectric impedance method was employed to measure the resonance frequency change with the rock bolt pre-tension looseness, and the relationship between the extent of the pre-tension degradation of the rock bolt and the resonance frequency of the specimen was built. In addition, based on the root mean square deviation (RMSD) method, a normalized rock bolt pre-tension looseness index is proposed here to evaluate rock bolt pre-tension looseness.

## 2. Principles

### 2.1. Electro-Mechanical Impedance

**Figure 1:**
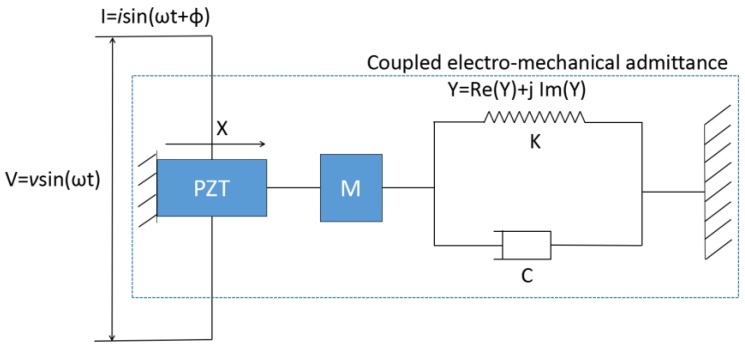
1-D model for the lead–zirconate–titanate (PZT)-drive dynamic structural system.

Electro-mechanical impedance is a novel method for monitoring local damage in a structure. The one-dimensional model of electro-mechanical impedance theory was first proposed by Liang et al. [28]. A simplified illustration of an integrated PZT and host structure system is shown in Figure 1. In this model, one end of the PZT is fixed and the system is considered a single degree-of-freedom mass-spring-damper system. Under the input of V = *v*sin(*ω*t), the impedance of the coupled system is affected by the dynamics of the PZT and the adjacent area of the host structure.

In this system, the electrical admittance *Y*(*ω*), which represents the inverse of the electrical impedance, is shown as

(1) $$Y(\omega )=j\omega \frac{wl}{h}\left[\frac{Z_{A}(\omega )}{Z_{A}(\omega )+Z_{S}(\omega )}\frac{d_{31}^{2}{{\bar{Y}}_{11}}^{E}\tan(\kappa l)}{\kappa l}+{\bar{\varepsilon }}_{33}^{T}-d_{31}^{2}{{\bar{Y}}_{11}}^{E}\right]$$

where *Z_A_* and *Z_S_* represent the PZT’s and structure’s mechanical impedance, respectively. In addition, *ω* is the excitation frequency, ρ is the density of the PZT, *l* is the PZT length, *w* is the PZT width, *h* is the PZT thickness, κ is the wave number (=$\omega /c_{t}^{E}$), and $c_{t}^{E}$ is wave velocity (=$\sqrt{{\bar{Y}}_{11}^{E}/\rho }$). *d*_31_ is the piezoelectric constant in the *x*-direction at zero stress, ${\bar{\varepsilon }}_{33}^{T}$=$\varepsilon_{33}^{T}$(1 − δj) and ${\bar{Y}}_{11}^{E}$=$Y_{11}^{E}$(1 + η*j*), where η and δ are the mechanical loss factor and dielectric loss factor, $\varepsilon_{33}^{T}$ is the complex dielectric constant of piezoelectric material under zero stress; $Y_{11}^{E}$ is the complex modulus in the *x*-direction under zero stress. Based on this formula, the change of electrical impedance of host structure can be detected through the bonded PZT. With a high frequency excitation, very small damage can be detected.

### 2.2. Root-Mean-Square Deviation Based Damage Index

In this study, root mean square deviation (RMSD) was applied to quantify the severity of pre-tension looseness. The RMSD method, previously used by Giurgiutiu et al. [29], has the following expression:

(2) $$\rho_{RMSD}(\%)=\sqrt{\frac{\sum_{i=1}^{i=N}{\left(y_{i}-x_{i}\right)}^{2}}{\sum_{i=1}^{i=N}{\left(x_{i}\right)}^{2}}}\times 100$$

where *y_i_* and *x_i_* represent the electrical impedance or electrical admittance before and after the damage, respectively. Since the electrical impedance and electrical admittance are complex, Equation (2) can be further expressed as

(3) ρRMSDR(%)=∑i=1n(Re(yi)−Re(xi))2∑i=1n(Re(xi))2×100

(4) ρRMSDI(%)=∑i=1n(Im(yi)−Im(xi))2∑i=1n(Im(xi))2×100

where $\rho_{RMSDR}$ and $\rho_{RMSDI}$ are the real part and the image part of the electrical impedance or electrical admittance before and after the damage. It should be noted that the imaginary part $\varepsilon_{33}^{T}$ is more sensitive to the temperature variation than the real part since the dielectric constant is temperature-sensitive and only affects the imaginary part. Therefore, the real part of the admittance (or impedance) is mainly used for monitoring in applications [30].

In order to quantify the looseness degree of the pre-stress on a rock bolt, the normalization of RMSD of the real part of electrical impedance is proposed as the looseness index:

(5) $$I_{RMSDR}^{i}=\frac{\rho_{RMSDR}^{b}-\rho_{RMSDR}^{i}}{\rho_{RMSDR}^{b}-\rho_{RMSDR}^{t}}$$

where the *i* is the *i*th sequence number of experiment, and $\rho_{RMSDR}^{b}$ and $\rho_{RMSDR}^{t}$ are the RMSD values without looseness and with total looseness, respectively. In this paper, this damage index is used to evaluate rock bolt looseness or the loss of the pre-stress.

## 3. Experimental Tests

### 3.1. Smart Washer and Test Specimen

Piezoceramic materials, which have direct and converse piezoelectric effects, are often used to build transducers. With the direct piezoelectric effect, a piezoceramic transducer can produce an electric charge when stress is applied, and the opposite process is called the converse piezoelectric effect.

**Figure 2:**
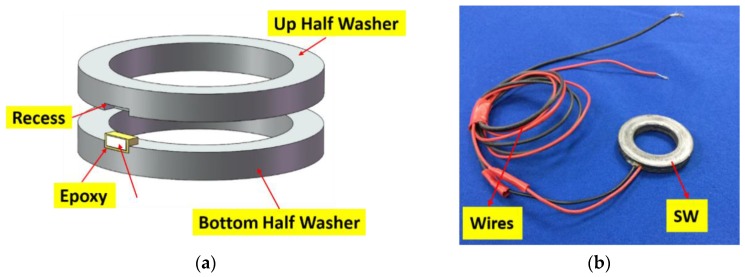
The design of the smart washer with connecting wires: (**a**) schematic diagram and (**b**) photograph.

To enable easy use of piezoceramic materials in a rock bolt with proper protection, a PZT-based device, the smart washer, fabricated by sandwiching a piezoceramic transducer between two flat metal rings, as shown in Figure 2, was adopted.

**Figure 3:**
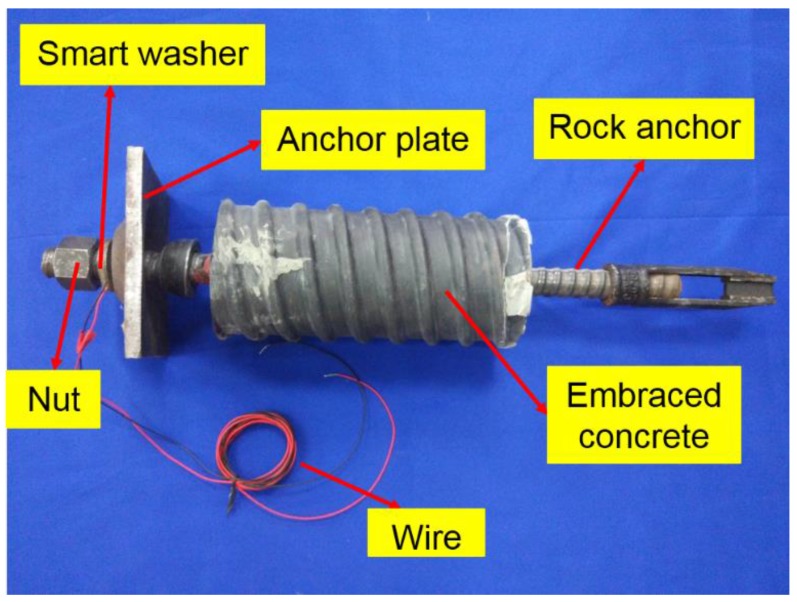
Detailed information of the rock bolt specimen.

In this study, the smart washer was mounted on a specimen, which consisted of a rock anchor, a heavy hex nut, a smart washer, and a bearing anchor plate. Detailed information of the specimen is shown in Figure 3.

### 3.2. Instrumentation Setup

**Figure 4:**
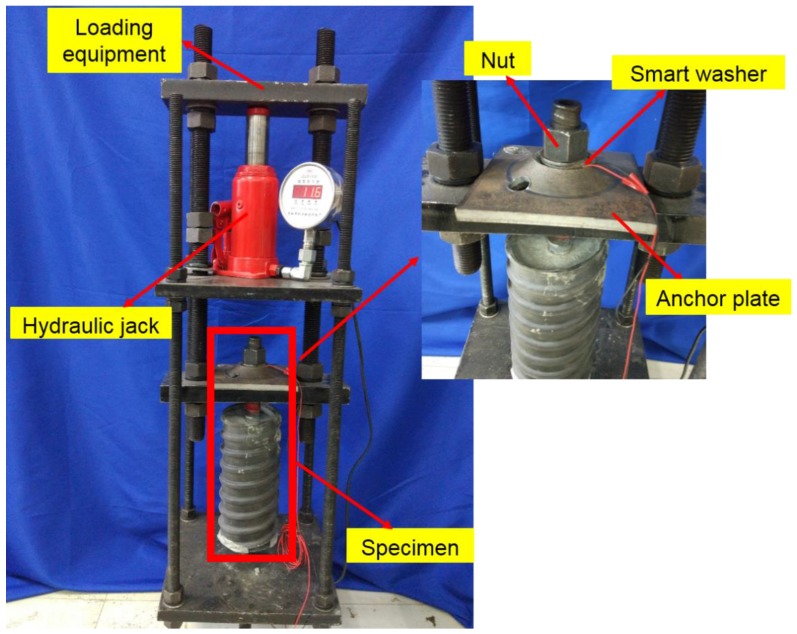
The loading system of rock bolt specimen.

**Figure 5:**
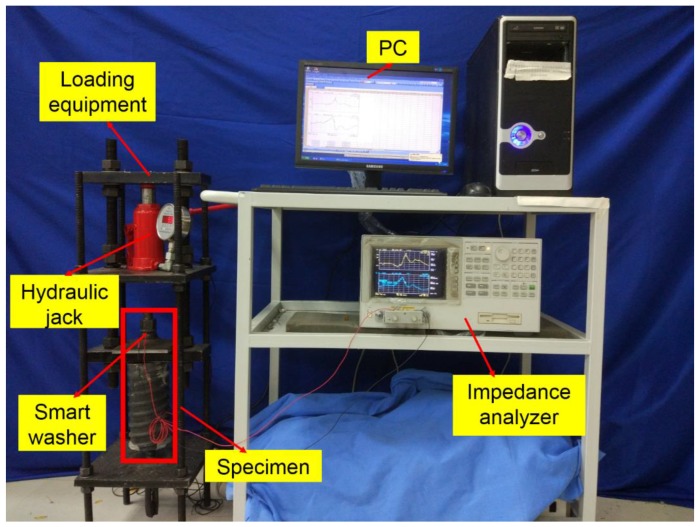
The entire experimental setup.

Smart washer-based rock bolt pre-tension monitoring using piezoelectric impedance measurement consists of three parts: a hydraulic jack, a loading frame, and an electrical impedance measuring system. The specimen fabrication is shown in Figure 4 and the experimental setup is shown in Figure 5.

In the experiment, the pre-tension or the pre-stress of rock bolt was controlled by the hydraulic jack with a range of 0–30 MPa. The loading procedure that consisted of eleven loading cases from 30 MPa to 0 MPa was carried out, as listed in Table 1. The decrease of the stress or the tension of the rock bolt simulates the process of the loss of pre-stress, the loss of pre-tension, or the looseness of the rock bolt. For example, the case of 0 MPa indicates the total looseness of the rock bolt.

### 3.3. Test Procedures and Frequency Range

**Figure 6:**
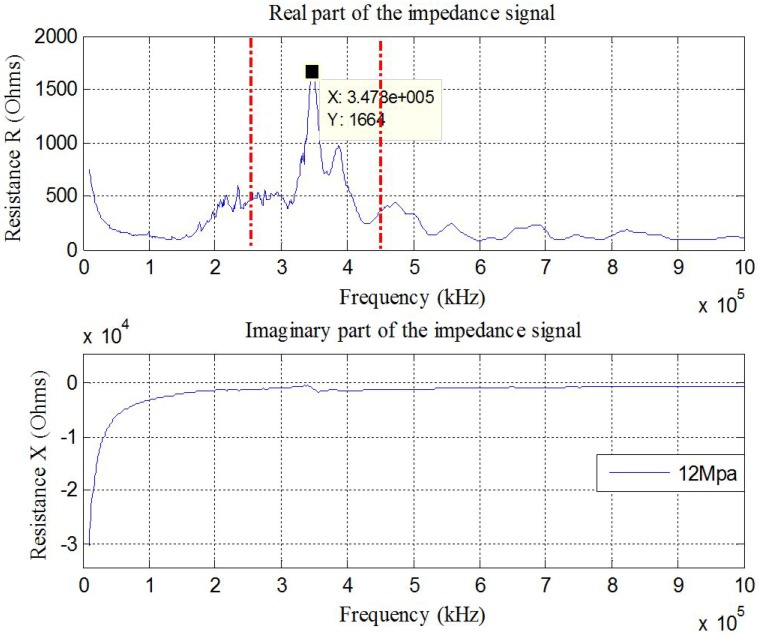
Electrical impedance signature acquired from the PZT patch (10 kHz–1 MHz).

The smart washer impedance test procedures were carried out in two steps:

(1) The wide frequency range test: To detect the incipient-type damage, the wavelength of the excitation signal should be less than the characteristic length of the damage. With high frequency excitation when the wavelength is much smaller than that of the damage, it is easy to detect the change in structural integrity. Excitation of 10–1000 kHz with 801 sampling data points was used with a pre-stress of 12 MPa in this experiment, as shown in Figure 6.

(2) The narrow frequency range test. From Figure 6, there is a sharp peak around 347.8 kHz between the red dotted lines in the real part of the electrical impedance signature. According to the Sauerbrey equation [31], the frequency shifts in the dominating peaks of the impedance signature of the PZT patch are proportional to the square of its fundamental resonance frequency. It is expected that some changes of the mechanical properties of the host structure may have caused some significant variation in the resonance frequency shifts of the electro-mechanical impedance functions of the PZT patch. To find the change in peak frequency value, a narrow frequency range from 250 kHz to 450 kHz was used.

## 4. Experimental Results and Analysis

**Figure 7:**
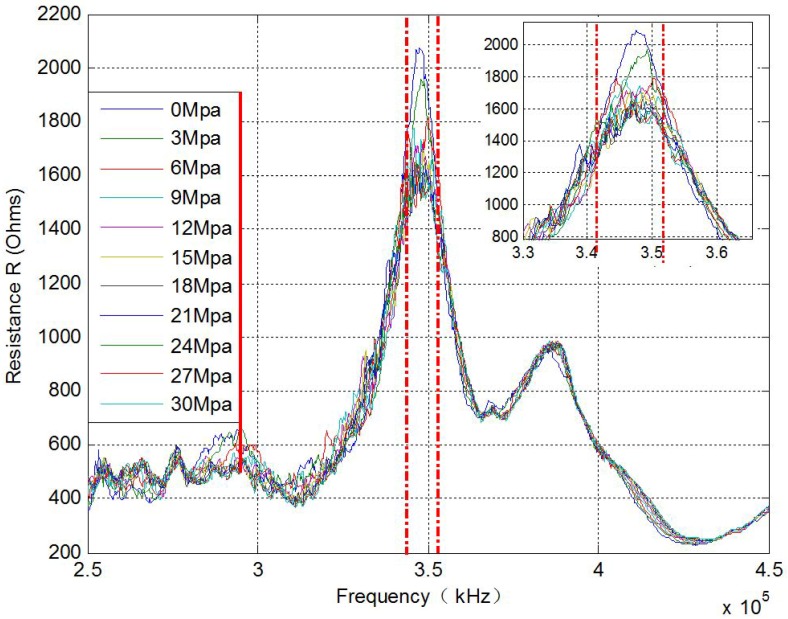
Electrical impedance signature of real part acquired from the SW (250 kHz–450 kHz).

According to Table 1, 11 different loading scenarios were investigated with the pre-stress level reduced from 30 MPa to 0 MPa. For each loading case, the electrical impedance signature of the PZT transducer was directly acquired by the impedance analyzer. The real parts of the electrical impedance for the eleven tests over the frequency range of 250 kHz to 450 kHz are shown in Figure 7. There is a frequency shift at the peak frequency in Figure 7. However, with the development of the loss of the pre-stress, the dominating frequency peak shifts back and forth, and the dominating frequency peak cannot be used as an index to indicate pre-stress change.

**Figure 8:**
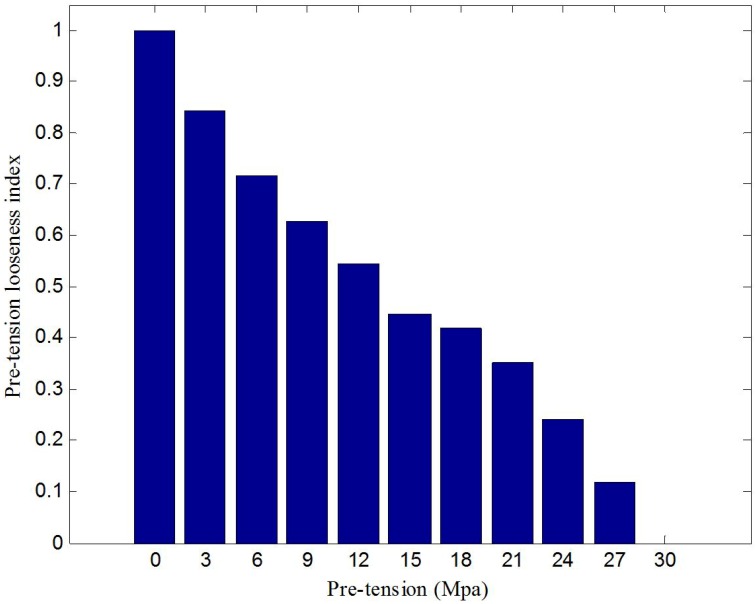
Normalized RMSD-based rock bolt looseness index from the first experiment.

**Figure 9:**
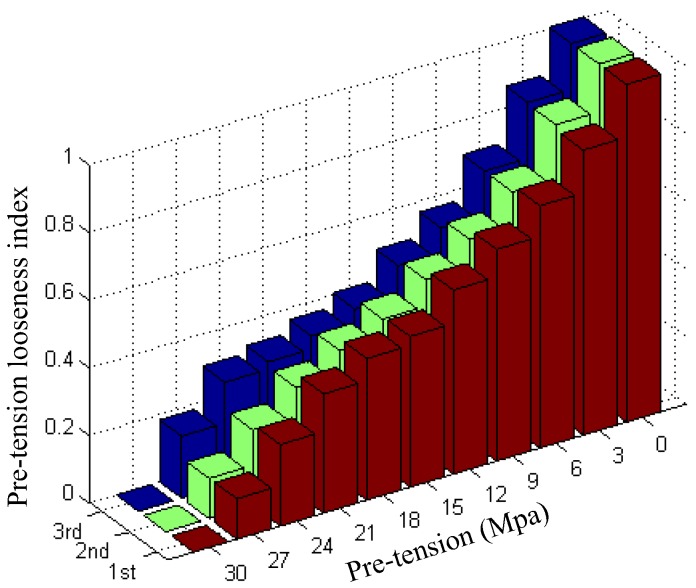
The normalized RMSD-based rock bolt looseness indices of three repeated experiments.

To reveal the looseness quantitatively, the normalized RMSD-based rock bolt looseness index was used. With the help of Equation (5), the processed result is shown in Figure 8. The bolt looseness index is 0 in the case of 30 MPa pre-tension (without the loss of any pre-stress); in the case of a rock bolt load of 0 MPa (completely looseness), the rock bolt looseness index is 1. The indexes clearly show that bolt looseness increases with reductions in applied torque. Figure 9 shows the experimental results of three repeated experiments, which validate the reliability and repeatability of the proposed looseness index. It is clear that the rock bolt pre-tension looseness index can effectively reflect the severity of the rock bolt looseness.

## 5. Conclusions

In this paper, a piezoceramic smart washer is proposed to monitor the pre-load or pre-stress loss of a rock bolt. The authors propose a robust and feasible rock bolt looseness monitoring approach, which is based on the electro-mechanical impedance method. A rock bolt specimen with a smart washer was investigated under different pre-stress levels. Due to a structural stiffness reduction, the frequency of the real part of the electrical impedance signature increased with the decrease in pre-stress levels. Furthermore, a normalized RMSD bolt looseness index was applied to show the severity of rock bolt pre-stress loss, and the proposed method successfully monitored the looseness level of the rock bolt, as demonstrated experimentally. Future work will involve field testing of the proposed method, with more testing sets to assess the feasibility of the proposed smart washer and looseness index.

## Figures and Tables

**Table 1:** Experimental procedure with eleven loading cases.

Sequence Number	1	2	3	4	5	6	7	8	9	10	11
Pre-load (MPa)	30	27	24	21	18	15	12	9	6	3	0
